# Combined nifuroxazide and SAT05f therapy reduces graft-*versus*-host disease after experimental allogeneic bone marrow transplantation

**DOI:** 10.1038/cddis.2016.399

**Published:** 2016-12-01

**Authors:** Huijie Jia, Tiesuo Zhao, Yinghua Ji, Xiaolong Jia, Wenjing Ren, Chen Li, Minming Li, Yali Xiao, Hui Wang, Kailin Xu

**Affiliations:** 1 Department of Pathology, Xinxiang Medical University, Xinxiang 453000, Henan, China; 2 Research Center for Immunology, School of Basic Medical Sciences, Xinxiang Medical University, Xinxiang 453003, Henan, China; 3 Department of Oncology, The First Affiliated Hospital of Xinxiang Medical University, Xinxiang 453000, Henan, China; 4 Department of Dermatology, The First Affiliated Hospital of Xinxiang Medical University, Xinxiang 453000, Henan, China; 5 Laboratory of Transplantation and Immunology, Xuzhou Medical University, Xuzhou 221002, Jiangsu, China

## Abstract

Acute graft-*versus*-host disease (aGvHD) is the major barrier to the broader use of allogenetic hematopoietic stem cells. However, currently these are no highly specific and efficient drugs. Monotherapy is not sufficient and more efficient and safe therapeutic regimen are urgent need. Studies demonstrated TLR9 and Stat3 signal pathways are critical for antigen-presenting cell maturation and T-cell activation, which are important mediators in aGvHD. Specific block these two critical signal pathways using their inhibitors SAT05f and nifuroxazide may be the novel strategies for aGvHD therapy. The results showed combined therapy significantly decreased the severity of aGvHD and prolonged the survival rate. Furthermore, after treatment, the activation of CD4^+^ effect T cells was reduced, whereas Treg cells was increased, and the cytokine release was inhibited. In conclusion, combined therapy of nifuroxazide with SAT05f may be potential for the prevention or treatment of aGvHD, providing theoretic and experimental basis.

Graft*-versus-*host disease (GvHD) is a potentially devastating complication with high morbidity and mortality in patients after allogeneic hematopoietic stem cell transplantation (HSCT).^1, 2^ In spite of great development has been made, the treatment progress of this disorder has been slow in recent years. Corticosteroids are standard fist-line therapy for GvHD, however, significant morbidity rates are even more than 40%.^2, 3^ As the complication and multistep in GvHD development, deep understanding of the mechanisms involved in the pathogenesis could yield novel therapeutic targets. The pathogenesis of GvHD can be conceptualized in three phages,^4^ in which the donor T cells activation in the second phase plays a center role. Therefore, the main strategies nowadays to prevent and treat GvHD are applications of agents deleted T cells or suppressed critical molecular pathways involved in T-cell activation and proliferation, including monoclonal antibodies,^5, 6, 7^ immunosuppressive drugs^8, 9, 10^ or cytotoxic drugs.^11, 12^ Although these new therapies have been proposed to treat GvHD, the outcome was still not satisfactory, and various side effects were seen.^13^ What's more, monotherapeutic strategy has failed to yield great clinical benefits,^14, 15^ thus apply combined agents that against multiple vital targets may achieve more effective control of GvHD.

The cytokines, such as IL-6, IL-21 and IL-23, that activate STAT3 are necessary for the onset in the second phase of acute GvHD (aGvHD)^16, 17, 18^ and the prolonged activation of STAT3 was found in donor T cells and GvHD-targeted organs.^19^ In contrast, murine recipients of allo-BMT with CD4^+^ T cells lacking STAT3 did not exhibit the typical symptoms of GvHD and exhibited conspicuously persistent survival^20^ illustrating that STAT3 signaling played a critical role in the activation and maturation of CD4^+^ T cell during aGvHD.^21, 22, 23^ Furthermore, knockout of SOCS3, the negative regulator of the JAK2/Stat3 signaling pathway, has been shown to markedly increase the incidence of aGvHD.^24^ Correspondingly, blockade of the upstream signaling pathway of STAT3, such as JAK2 or IL-6, using the specific antagonists or antibodies, have showed protective effects against GvHD in several murine models.^25, 26, 27, 28^ Nifuroxazide was initially used as an intestinal broad-spectrum antibiotic and has been demonstrated that it could specifically inhibit STAT3 phosphorylation by suppressing the JAK family kinases Jak2 and Tyk2, and caused a decrease in viability of myeloma cells recently.^29^ Additionally, no cytotoxic effect of nifuroxazide has been showed by experimental and clinical evidences, illustrating the security in clinical practice.^30, 31^

Increasing evidences from experimental HSCT suggested that conditioning-mediated tissue damage also played an important role in initiating and amplifying GvHD by propagating the cytokine storm characteristics.^32^ As the activation of CpG motif in TLR9 plays a vital role in the first phase via inducing inflammatory cytokine and activating antigen-presenting cell (APC) involved in innate and adaptive immunity, it would be an interesting target for the treatment of GvHD.^33, 34^ Previous studies have showed that treatment with TLR9 agonistic CpG ODN (cytosine-phosphate-guanine oligodeoxynucleotide) aggravated GvHD lethality in the mouse model^35, 36^ on the contrary, deficiency in TLR9 could lead to increasing of GvHD survival.^37, 38^ SAT05f, an inhibitory ODN of TLR9 inhibitor, have been exhibited great protective effects in various mouse models of immunologic diseases.^39, 40, 41^ In this study, we wondered that if a combination of nifuroxazide and SAT05f therapy would improve curative effect and prognosis in a murine model of aGvHD.

## Results

### Combined of nifuroxazide with SAT05f markedly decreased severity and prolonged the survival rate from aGvHD

**Figure 1:**
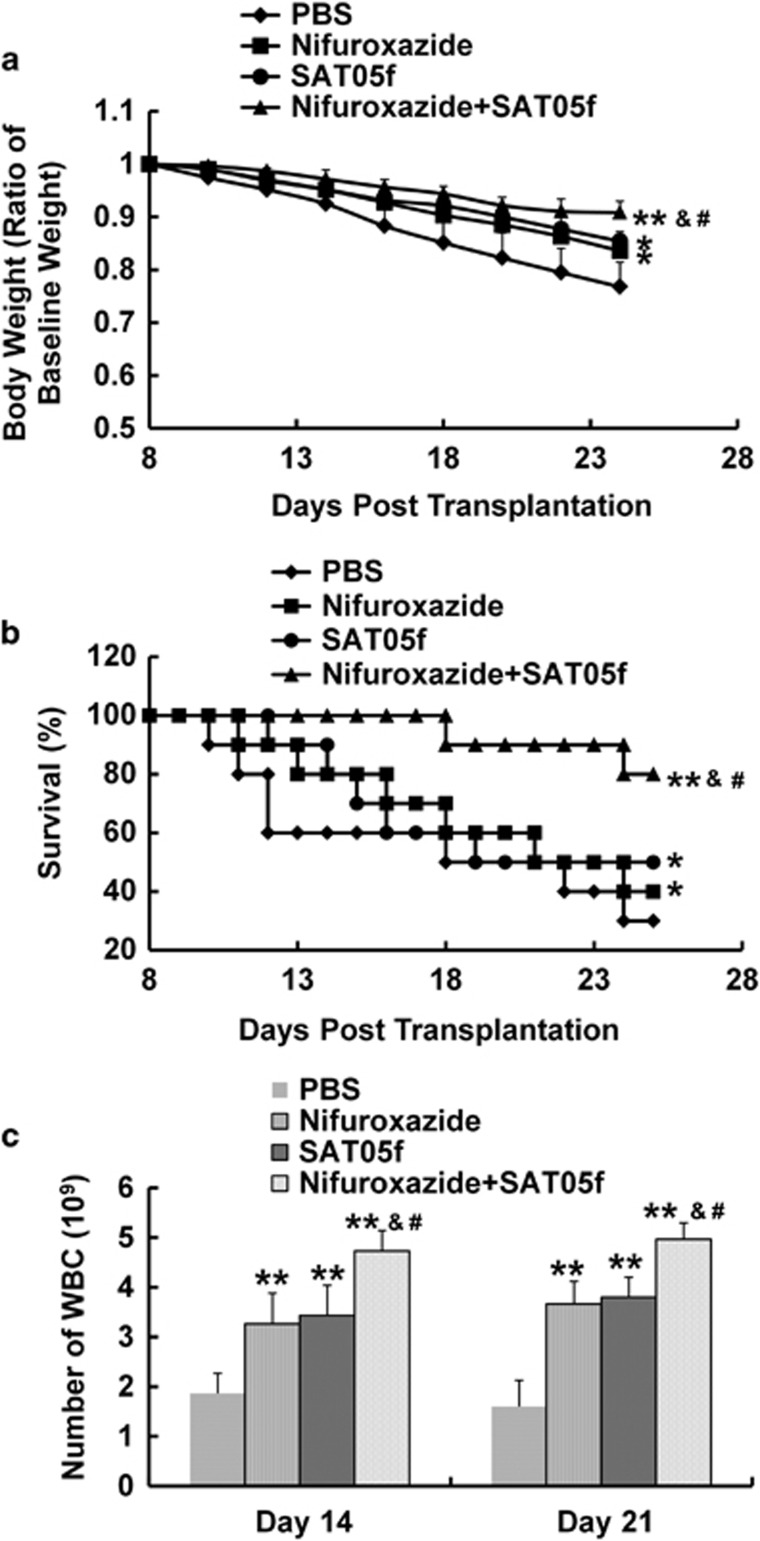
Nifuroxazide plus SAT05f ameliorated aGvHD severity. (**a**) The body weight change of mice in each group (represent the ratio of baseline weight). (**b**) The survival rate of mice in every group after different treatment. (**c**) WBC count from peripheral blood. Results are expressed as means±S.D. *P<0.05 *versus* PBS group, **P<0.01 *versus* PBS group, ^&^*P*<0.05 *versus* nifuroxazide group, ^#^*P*<0.05 *versus* SAT05f group. All experiments were done with each group of eight mice

Body weight changes of mice in each group after different treatment were shown in Figure 1a. After total body irradiation (TBI), all the recipient mice showed sharp body weight loss in the first week and never started to make a weight again until death (data not shown). The average body weight of mice in PBS-treated group was heavily decreased more than that in the two single treatment groups and the combination group (Figure 1a). It was noteworthy that the body weight of mice treated with combined nifuroxazide and SAT05f was less decreased than that treated with either the nifuroxazide or SAT05f (Figure 1a), whereas there is no statistic difference of body weight change between the two single treatment group. In addition, treatment of aGvHD with either nifuroxazide alone or in combination with SAT05f prolonged the survival time of the recipient mice compared with treatment with PBS (Figure 1b). Moreover, throughout the entire 25 days observation, the mean survival time of mice in the combination treatment group was longer than either of the monotherapy group (Figure 1b). Nevertheless, there was no significant difference of the mean survival time between the nifuroxazide group and the SAT05f group. Furthermore, the white blood cell (WBC) count was tested to detect the hematopoietic reconstruction. The total number of WBCs was significantly decreased after TBI and gradually increased after transplantation. The nifuroxazide plus SAT05f treatment showed a significant WBC gain than the other three groups (Figure 1c), and the single treatment with nifuroxazide or SAT05f also showed WBC restore than PBS-treated group.

### Combination therapy with nifuroxazide and SAT05f significantly decreases histopathologic damage of aGvHD target organs in mice with aGVHD

**Figure 2:**
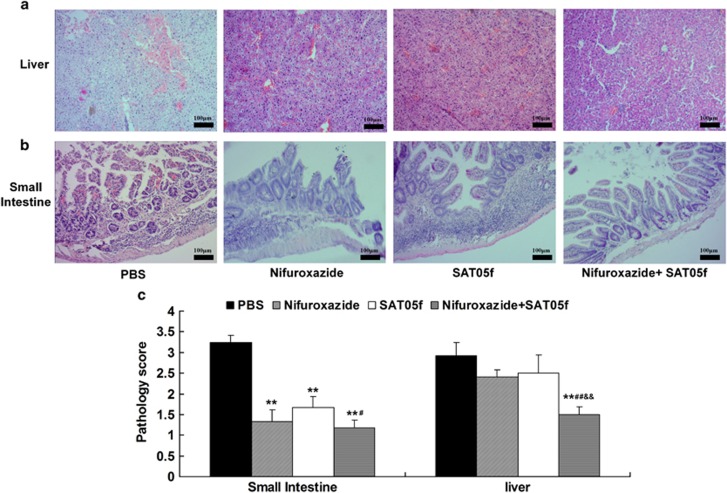
Combination treatment of STAT3 and TLR9 inhibited GvHD target tissue. Recipients of BALB/c with allo-BMT were treated with nifuroxazide or/and SAT05f per dose daily on day 4 after BMT. On day 14, the mice were killed and liver and small intestine were collected, processed and stained with HE. Hispathology of the target tissues in the mice with aGvHD after different treatment methods. (**a**) Hispathology of liver. (**b**) Hispathology of small intestine. (**c**) Semiquantitative histological analysis of at least five examined recipient mice in each organ. Original magnification × 100. **P<0.01 *versus* PBS group, ^&&^*P*<0.01 *versus* nifuroxazide group, ^#^*P*<0.05 *versus* SAT05f group, ^##^*P*<0.01 *versus* SAT05f group

After 2 weeks from transplantation, PBS-treated mice exhibited high degree edema, hepatic congestion and even necrosis of hepatocyte in liver, whereas mice in the monotherapy group showed low degree edema, hepatic congestion and the necrosis scarcely seen. When compared with the combination group, the less injury and the better liver morphology were seen than the other three groups (Figure 2a). In addition, the small intestines from mice with vehicle group have severe blunting of villi, glandular organ rupture and an inflammatory infiltrate. Less villous blunting and fusion were showed in the nifuroxazide and SAT05f-treated group. Significant restoration of the small intestinal villous architecture with little inflammatory infiltration was seen in the combination group (Figure 2b).

### STAT3 or TLR9 expression were corresponding inhibited after injection with nifuroxazide or SAT05f

**Figure 3:**
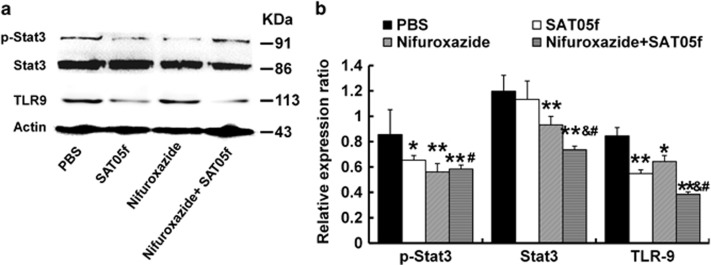
The STAT3 and TLR9 expression after different treatment. The lysates of livers from every group were separated by SDS-PAGE and subjected to immunoblotting. (**a**) Expression of STAT3, p-STAT3 and TLR9. (**b**) Relative expression for these proteins. *P<0.05 *versus* PBS group, **P<0.01 *versus* PBS group, ^&^*P*<0.05 *versus* nifuroxazide group, ^#^*P*<0.05 *versus* SAT05f group. Data are cumulated with the results from three independent experiments

Immunoblotting was taken to assess the expression of Stat3 and TLR9. The results showed that both monotherapy of nifuroxazide and combined therapy inhibited Stat3 and p-Stat3 expression. Subsequently, we measured TLR9 expression and found that treatment with SAT05f or combination of nifuroxazide and SAT05f inhibited TLR-9 expression, respectively, (Figure 3).

### Combination of nifuroxazide with SAT05f treatment reduced the activation of T lymphocytes and increased the ratio of regulatory T cells

**Figure 4:**
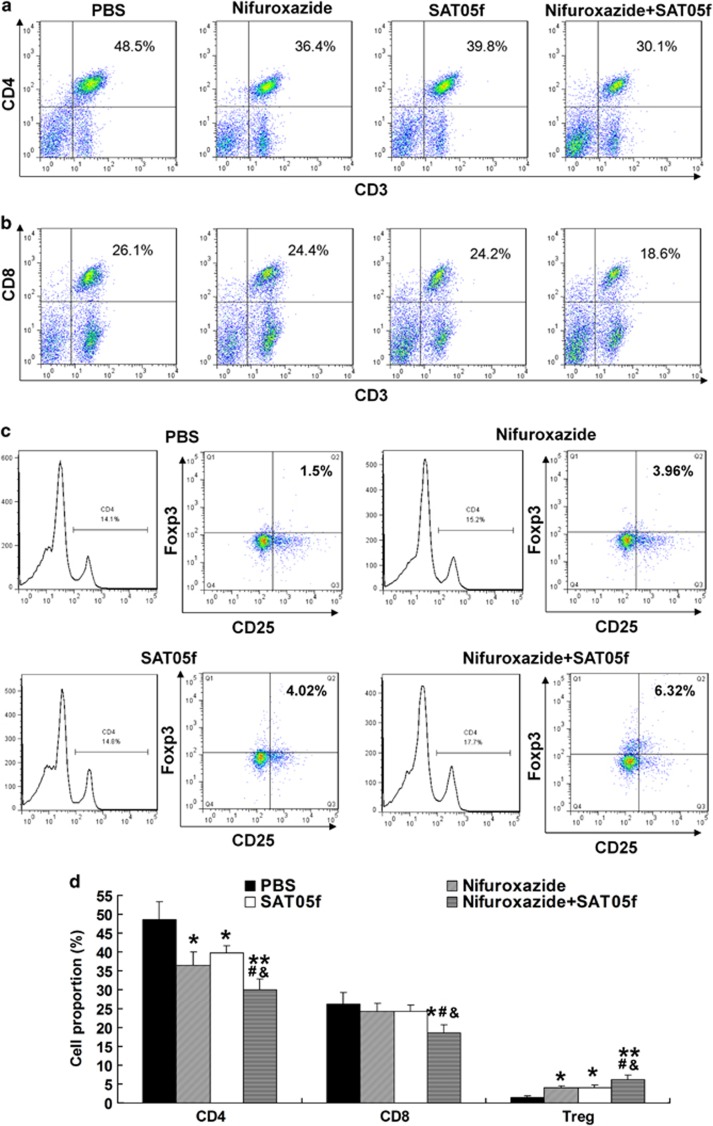
Combination therapy regulated T-cell differentiation after allogeneic BMT. On day 14 after transplantation, splenocytes were collected, determined with an automated cell counter, stained with appropriate T cells antibodies and quantified by flow cytometry. (**a**–**c**) The level of CD4, CD8 and Treg was evaluated in splenic T lymphocytes. (**d**) The percentage of T cells was analyzed (*n*=5). *P<0.05 *versus* PBS group, **P<0.01 *versus* PBS group, ^&^*P*<0.05 *versus* nifuroxazide group, ^#^*P*<0.05 *versus* SAT05f group

We evaluated whether the combination treatment altered the relative proportions of CD3^+^/CD4^+^ and CD3^+^/CD8^+^ splenic T cells. The results showed that combination treatment suppressed the populations for both the CD4^+^ and CD8^+^ T cells compared with that in the other three groups (Figures 4a and b), and the monotherapy with nifuroxazide and SAT05f inhibited CD4^+^ and CD8^+^ T cells compared with that in the PBS-treated group, whereas the percentage of T cells was similar among the nifuroxazide and SAT05f-treated group (Figures 4a and b). Previous researches have indicated that regulatory T cells (Treg) play an important role in suppressing the development of GvHD.^42^ Moreover, the CD4^+^/CD25^+^ Treg cells were further analyzed. The population of CD4^+^/CD25^+^ Treg cells in the combination groups was increased significantly when compared with that in the other three groups, whereas the numbers of CD4^+^/CD25^+^ Treg cells in nifuroxazide and SAT05f-treated group reminded at a higher lever than that in the PBS-treated group (Figure 4c). This compartment illustrated the greatest increment of Treg expression possibly in response to T cell inhibition for both CD4^+^ and CD8^+^ T cells.

### Nifuroxazide plus SAT05f treatment significantly inhibited cytokine release after allogeneic BMT

**Figure 5:**
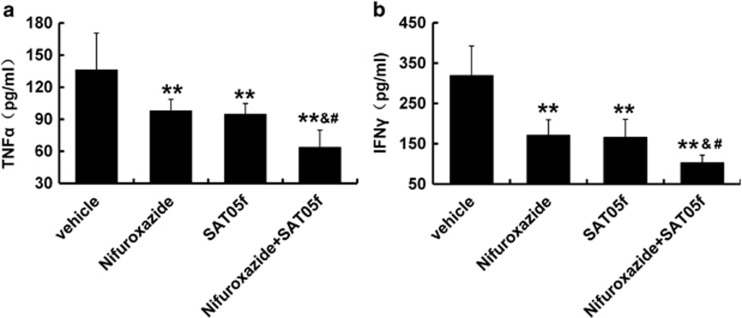
Nifuroxazide and SAT05f treatment effectively reduced serum cytokine levels in recipient mice with aGvHD. Mice were bled on day 14 after BMT and were analyzed for the TNF-*α* and IFN-*γ* cytokines. (**a**) TNF-*α* level in each group. (**b**) IFN-*γ* level in each group. *P<0.05 *versus* PBS group, **P<0.01 *versus* PBS group, ^&^*P*<0.05 *versus* nifuroxazide group, ^#^*P*<0.05 *versus* SAT05f group. Data are cumulated with the results from three independent experiments

It has been revealed that the ‘cytokine storm' fuels GvHD pathogenesis and in particular, TNF-*α* and IFN-*γ* were shown to play important role in determining the severity of aGvHD.^43, 44, 45^ Combination of nifuroxazide with SAT05f significantly suppressed serum levels of the two proinflammatory cytokines compared with the other three groups (Figure 5). Furthermore, compared with PBS-treated allo-recipients, the nifuroxazide or SAT05f-treated mice also showed cytokine release inhibition (Figure 5). Therefore, we reasoned that the protection from aGvHD might be the consequence of the inhibition of proinflammatory cytokine secretion by nifuroxazide.

## Discussion

The induction of aGvHD is a direct consequence of the donor T cell recognizes and responses to the host alloantigens, and STAT3 has been recognized to play a vital role in T cells activation during the pathogenesis of aGvHD. However, the progress of aGvHD involves multisteps and extremely complicated. Once donor T cells activated, the injury induced by aGvHD could hardly be reversed.^46^ Therefore, it is probable that an early step should be blocked to enhance the treatment of T-cell inhibition. The initiation of aGvHD stems from original conditioning regimen and accompanying intestinal tract damage, leading to the abundant release of inflammatory cytokines and TLR ligands, which act as an immune adjuvant on subsequent crucial host APC-donor T-cell interaction. Recent studies point toward the important role of TLRs-mediated sensing of bacterial DNA in the initiation and the aggravation of aGvHD.^35, 36, 37^

Several TLRs have been point toward the important role in the initiation and progress of GvHD.^43^ However, recent research described that during these TLRs, the activation of the TLR9-mediated sensing of bacterial DNA might be the most key factor in initiation of GvHD.^33, 34, 35, 37^ TLR9 downstream signaling activated a complicated response cascade, leading to host resistance by inducing inflammatory cytokine storm as well as enhancing antigen presentation by APCs,^43^ which was the critical target of the effector T cells in GvHD.^33, 35, 47, 48, 49^ As an inhibitory ODN, SAT05f is a TLR9 inhibitor and constitutes of CCT repeated in eight times.

In this study, we showed that the severity of aGvHD attenuation was potency since the combination of nifuroxazide with SAT05f decreased the histologic GvHD injury in liver and intestinal tract, as well as improved the survival of lethally irradiated mice. Moreover, the decrease of T-cell activation in spleen and infiltration in target tissues after treatment have been demonstrated in the study, illustrating that the protective effect is elicited by inhibiting donor effector T cells activation, migration and increasing Treg cells. In this context, the combined nifuroxazide with SAT05f therapy might synergistically affect T-cell function, in terms of inhibition of both proliferation and activation.

After transplantation, the activation of donor T cells permissive of the generation of cytokine storm, resulting in high levels of inflammatory factors, together with cytotoxic T cells, damage host target tissues. Thus we have developed an interest in analyzing the production of the proinflammatory cytokines IFN-*γ* and TNF-*α* after combination treatment. Alternatively, the release of both IFN-*γ* and TNF-*α* were reduced after treatment.

The immunoregulation network should be complex, crosstalk with each other and might be interplayed as a circle. TLR9 induces MyD88 dependent and independent pathways, resulting in activation of the several signal pathways, such as NF-*κ*B, and subsequent secretion of inflammatory cytokines such as IL-6, TNF-*α*, IFN-*γ* and so on,^43, 44, 45, 50^ leading to activation of host APCs. In response to such proinflammatory cytokines like IL-6,^51^ JAK2/STAT3 was activated, and subsequently mediated alloactivation of donor T cells by host APCs. Moreover, the pathway of IL-6/JAK2/STAT3 results in more secretion of proinflammatory cytokines, amplifying the immunologic injury.

**Figure 6:**
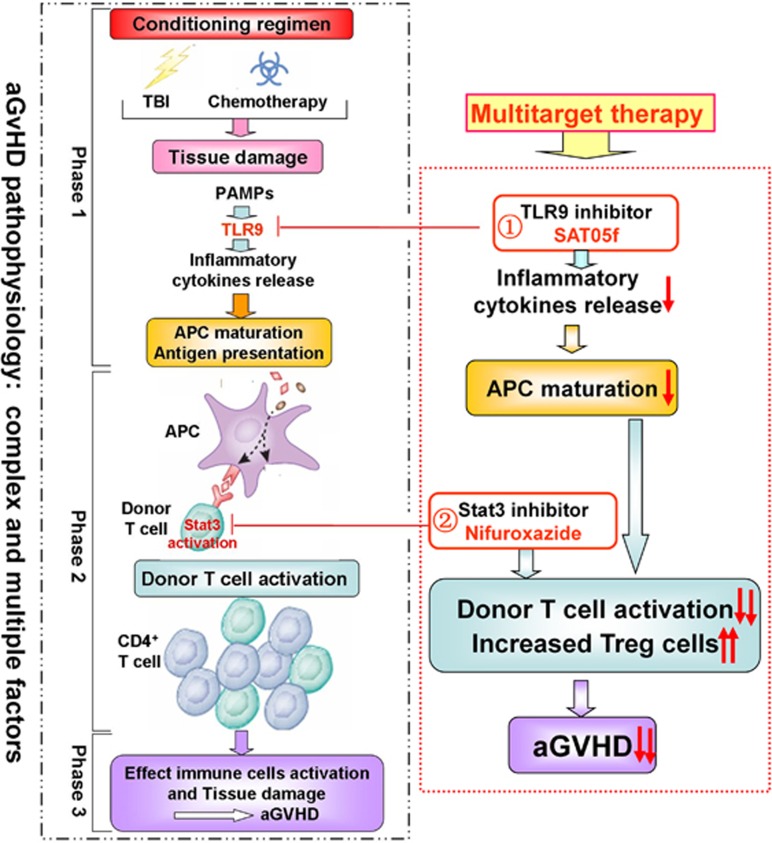
The multi-targeted STAT3 and TLR9 for aGvHD therapy. The pathogenesis of GvHD is complicated and can be conceptualized in three phages: host APCs activation by conditioning regimen; donor T cells recognize alloantigen and activation; finally allo-reactive donor T cells migrate and target tissues inducing organs injury. In these stages, donor T cells activation plays a center role in GvHD progression, in which the Stat3 played important role. In this study, application of nifuroxazide blocked Stat3 activation, and inhibited T cells activation and proliferation. In addition, alloactivate donor T cells by host APCs, TLR9 appears to be a special important cofactor in the development of GvHD. SAT05f inhibited TLR9 pathway in the initial stage of aGVHD progressing, preventing inflammatory cytokines secretion and then inhibiting the activation of T cells. Combined suppressing of these two signaling pathways synergetically intervened in multiple key signals in effect T-cell activation, and thus exhibited more effectively in deterring the occurrence of aGVHD

We illustrated in this study that the nifuroxazide combined with SAT05f would inhibit STAT3 and TLR9 activation, resulting in the decrease of production of proinflammatory cytokines, and would reduce donor T-cell response to host APC, and therefore lead to attenuated aGvHD (Figure 6). The data give new insight into the co-treatment of aGvHD with and nifuroxazide and other immunosuppressants, and suggest that combination therapy of nifuroxazide with SAT05f may be potential therapeutic drugs for the prevention or treatment of aGvHD.

## Material and methods

### Mice and reagents

Eight-week-old male C57BL/6 and BALB/c mice were purchased from Experimental Animal Center of Zhengzhou University (Zhengzhou, Henan, China). Nifuroxazide was obtained from Sigma (St. Louis, MO, USA), and was dissolved in DMSO. SAT05f, an inhibitory ODN used in this and previous studies, has a sequence of 5′-CCTCCTCCTCCTCCTCCTCCTCCT-3′ and was provided by Sangon Biotech (Shanghai, China).

### Induction and treatment of aGvHD

BALB/c mice were used as recipients and C57BL/6 mice were used as donors. Before allo-BMT, the recipients were fed with sterile food and acidifier water for 1 week. Briefly, recipients were irradiated with TBI (7.5 Gy, ^60^Co source). Four hours after TBI, these mice were injected intravenously with the mixture of 5 × 10^6^ bone marrow cells and 5 × 10^6^ splenocytes per mouse prepared from allogeneic donors via tail vein. For the treatment, the recipient mice were randomly divided into four groups (*n*=12). At day 4 after allo-BMT model, the mice in the monotherapy of nifuroxazide or SAT05f group were continued injected i.p. (intraperitoneal) with nifuroxazide (200 *μ*g/mouse) or SAT05f (10 *μ*g/mouse), respectively, for 1 week, and the mice in combination group were injected i.p. with both nifuroxazide and SAT05f, whereas the mice in the vehicle group were injected i.p. with PBS. Regularly, the peripheral WBC count was determined, the body weight changes, clinical behaviors and the survival time of aGvHD were recorded.

### Histology and immunoblotting

At the day 14 after transplantation, the mice were killed and the liver and small intestine were collected and then frozen in liquid nitrogen or fixed in 4% paraformal-dehyde immediately. Immunoblotting was performed as previously described.^52^ For immunoblotting, cell lysates were obtained after cell debris discarded. Proteins were separated in 12% SDS-PAGE gels, transferred to polyvinylidene difluoride (PVDF) membranes (Millipore Corp, Billerica, MA, USA) and immunoblotted with appropriate primary antibodies specific for total Stat3, p-Stat3, *β*-antin (Cell Signaling Technology, Danvers, MA, USA) or TLR-9 (Santa Cruz Biotechnology, Delaware Ave Santa Cruz, CA, USA). Sections (5 *μ*m) were subjected to standard hematoxylin and eosin (HE) staining.

### Flow cytometry

Spleens were collected 2 weeks after allo-BMT, passed through a 40-mm nylon cell strainer and then collected in PBS. RBCs were removed with Red Blood Cell Lysis Buffer (Beyotime Biotechnology, Shanghai, China). Cells were washed and resuspended at 1 × 10^7^ cells/ml in PBS. Aliquots (0.1 ml) were placed on ice and labeled with appropriate fluorochrome-labeled Anti-Mouse CD3, Anti-Mouse CD4, Anti-Mouse CD8 and Anti-Mouse CD25 (Biogems international, Westlake Village, CA, USA) for 30 min in the dark. Three-color staining was performed for detecting CD4^+^, CD25^+^ T cells and Treg cells using Mouse Regulatory T cell staining kit (Affymetrix, Santa Clara, CA, USA), according to the manufacturer's protocol. Stained cells were washed with iced PBS and resuspended in PBS containing 1% paraformaldehyde. The fluorescence intensity was measured with a dual laser benchtop flow cytometer (Guava easyCyte HT (EMD Millipore Corporation, USA)) with a minimum of 10 000 events collected.

### Measurement of serum cytokines

Cytokines concentration of TNF-*α* and IFN-*γ* in serum samples were detected using mouse enzyme-linked immunosorbent assay (ELISA) kits (Raybiotech, Norcross, GA, USA) according to the experimental procedure.

### Statistical analysis

Data were calculated by the GraphPad Prism 4.0 software and were presented as the means±S.D. of at least three independent experiments. The methods including Mann–Whitney *U*-test, log-rank test, Student's *t-*test or one-way ANOVA was used when appropriate. In all experiments, *P*<0.05 was considered to be statistical significance.
